# Progressive Post-laminectomy Adult Spinal Deformity: A Case Study With a 24-Year Imaging Span

**DOI:** 10.7759/cureus.116757

**Published:** 2026-09-22

**Authors:** Michael T Young, Coral Stone

**Affiliations:** 1 Pain Management, Adventist Health Ukiah Valley, Ukiah, USA; 2 Pain Management, Menlo-Atherton High School, Menlo Park, USA

**Keywords:** adult spinal deformity, cobb angle, de novo degenerative scoliosis, lumbar facet injection, lumbar spondylolisthesis, post-laminectomy syndrome

## Abstract

Adult spinal deformity (ASD) arising after remote lumbar decompression is a recognized but under-reported clinical entity, and its long-term radiographic trajectory in conservatively managed patients is incompletely characterized. We describe a 56-year-old female with chronic axial low back pain who underwent lumbar surgery in November 2001 (presumed decompressive laminectomy) and for whom imaging is available from a standing preoperative radiograph obtained in April 2001 through nine years of serial standing studies obtained between 2016 and 2025. The preoperative film demonstrated no significant lumbar coronal deformity, although its limited field of view could not exclude a thoracic curve. Magnetic resonance imaging in 2014 demonstrated a grade 1 L4-L5 degenerative spondylolisthesis with severe facet arthropathy and mild spinal stenosis. Standing long-cassette radiographs over the subsequent nine years documented progression of a lumbar coronal curve from 35° to 41-43° and the development of a compensatory thoracic curve increasing from 15° to 31°, evolving into a severe S-shaped deformity. The patient was treated initially with a lumbar facet block and ongoing multimodal conservative care. Measurement variability across radiologists, driven by inconsistent end-vertebra selection, limits the strict comparability of these angles and is an under-recognized confounder in real-world deformity surveillance. The case illustrates the tempo of post-laminectomy degenerative scoliosis, highlights the need for standardized standing long-cassette imaging protocols in symptomatic adults with prior decompression, and frames the conservative versus reconstructive decision in light of contemporary ASD evidence.

## Introduction

Adult spinal deformity is increasingly common with an aging population, with prevalence estimates of any spinal deformity reaching tens of millions of older adults [1]. A substantial proportion of these deformities develop de novo in mid-to-late adulthood through asymmetric degeneration of the lumbar motion segments [2,3], a process distinct from idiopathic scoliosis carried forward from adolescence. The natural history is one of slow but heterogeneous progression, with mean annual Cobb angle increase reported at approximately 0.83 ± 1.1°/year in a cohort of non-surgically managed adult deformity patients [4] and with evidence linking progression to advancing intervertebral disc degeneration and lateral vertebral translation [5].

Superimposed on this background, prior lumbar decompressive surgery is a recognized contributor to delayed segmental instability and iatrogenic spondylolisthesis [6,7]. Resection of posterior elements can compromise facet integrity and segmental stability, and long-term cohort data show that progressive anterior subluxation follows decompression in a substantial minority of patients, with incidence rising with the number of levels decompressed and the extent of bone removal [8]. Whether this same mechanism contributes to the development or acceleration of de novo coronal deformity later in life is less well characterized, particularly in patients followed conservatively over many years. A specific evidential difficulty is that, in our experience, preoperative imaging is seldom retrievable decades later, so the coronal alignment that preceded the index decompression is often impossible to establish.

We present a case study spanning 24 years, from a standing preoperative radiograph in 2001 to serial standing studies obtained between 2016 and 2025, in an adult woman with chronic axial back pain and progressive lumbar and thoracic deformity. The case highlights the tempo of post-laminectomy adult spinal deformity, the diagnostic imaging caveats that complicate longitudinal comparison, and the thresholds at which conservative care should be reconsidered.

## Case presentation

Patient background

Written informed consent for publication of this case report and the accompanying de-identified images was obtained from the patient and covers the 2001 preoperative radiograph and the disclosure of the surgical date.

A 56-year-old female presented in 2016 to a pain medicine clinic with a 27-year history of chronic axial low back pain. Pain was first reported in 1989 (at approximately age 29) and progressed over the subsequent decades. The patient underwent lumbar surgery in November 2001 at an outside institution. The original operative report is no longer retrievable given the interval since surgery. On the basis of the era and the patient’s subsequent radiographic findings of L4-L5 degenerative spondylolisthesis with severe facet arthropathy and central canal compromise, the procedure was most consistent with a decompressive lumbar laminectomy; the specific technique and levels are therefore inferred rather than documented, a limitation carried throughout this report. The patient was a former smoker, used alcohol intermittently (one to two times per month), denied substance use, and reported strong family support. Past medical history was notable for asthma (on inhaled fluticasone and rescue albuterol) and migraine (pro re nata (PRN) sumatriptan). Current pain medications included pregabalin 75 mg twice daily and naproxen 500 mg twice daily.

Presentation and examination

At the 2016 visit, the patient rated her pain at 6-8/10 on a visual analog scale. Pain was localized to the lower back with intermittent right-leg radiation, although she described it as “mostly localized.” It was worse in the morning, on standing, with sitting upright, leaning forward, and driving, and was relieved by reclining. The character was burning, constant, and at times stabbing. Prior non-pharmacological treatments, including physical therapy and acupuncture, had provided no durable relief.

On examination, she was alert and in no acute distress. Lumbar flexion and extension were limited, with tenderness over the lumbar facets and a positive facet-loading test. Motor strength was 5/5 in all major lower-extremity groups, sensation was intact to light touch, and deep-tendon reflexes were symmetric. Neurological examination was otherwise unremarkable. The combination of axial pain pattern, extension-rotation provocation, absence of radicular neurological deficit, and tenderness over the facet line was consistent with a facet-mediated pain syndrome on a background of degenerative deformity, although clinical findings alone are not diagnostic and larger studies have failed to reproduce the historical “lumbar facet syndrome” [9].

Imaging timeline

Preoperative Baseline (2001)

A standing anteroposterior lumbar radiograph obtained in April 2001, approximately seven months before the index procedure, was available (Figure 1). The study is a coned lumbar view encompassing approximately T12 to the sacrum rather than a full-length scoliosis series. On this film, the lumbar spine is grossly straight in the coronal plane, without the levoconvex curvature that characterizes the later studies, and the posterior elements appear intact at the visualized levels. No Cobb angle was reported at the time, and none has been measured retrospectively; the description here is the treating physician’s qualitative assessment of the film rather than a formal measurement.

**Figure 1:**
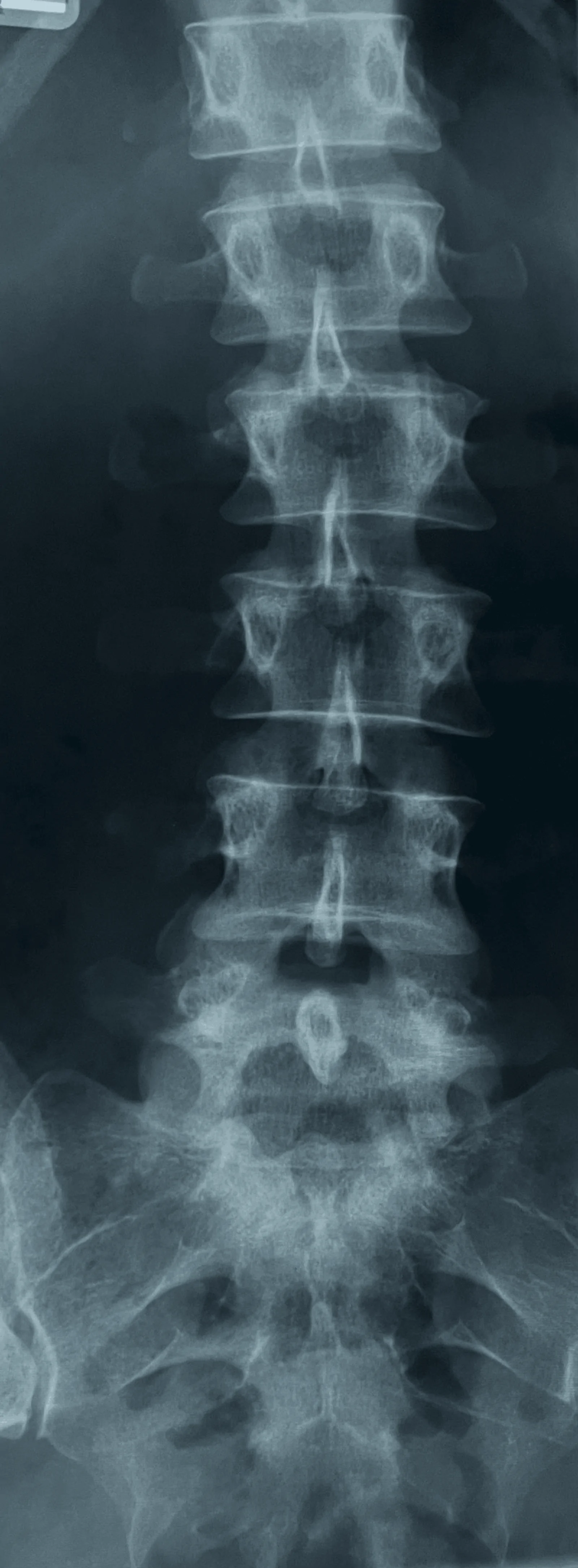
De-identified standing anteroposterior lumbar radiograph obtained in April 2001, approximately seven months before the November 2001 index procedure. The visualized field extends from approximately T12 to the sacrum; the thoracic spine is not included. The lumbar spine is grossly straight in the coronal plane, and the posterior elements appear intact at the visualized levels. Because this is a coned lumbar view rather than a standing long-cassette scoliosis series, curve magnitudes from this film are not directly comparable with those from the later standing scoliosis series.

Because this study is a coned lumbar view rather than a standing long-cassette series and because the thoracic spine is not visualized, it cannot be used to compute a thoracic curve or to establish sagittal or pelvic parameters, and it cannot exclude a coexisting thoracic curve. It nonetheless establishes that a significant lumbar coronal deformity was not present seven months before the index decompression.

Postoperative Imaging (2014-2025)

A lumbar magnetic resonance imaging (MRI) study performed in September 2014, approximately two years before she presented to the pain clinic, was available for review (Figure 2). The study was performed at 1.5 T with and without intravenous gadolinium contrast. It demonstrated anterior subluxation of L4 on L5 of 3.5 mm at midline (Figures 2A, 2B), with severe degenerative change at the L4-L5 facets, greater on the left, and hypertrophic change causing mild posterolateral encroachment on the dural sac; the anteroposterior dural-sac diameter measured 7.1 mm at this level. Severe degenerative change was also present at the left L5-S1 facet, and degenerative change at the left L3-L4 facet. The L2-L3 disc showed minimal broad-based bulging to the left of midline with a small left-sided annular tear. The remaining levels showed no encroachment on the dural sac, with dural-sac diameters of 12.2-13.9 mm and patent intervertebral foramina throughout (Figures 2C, 2D). Vertebral body signal was normal; there was no intradural lesion, no abnormal enhancement following contrast administration, and the conus terminated at the level of T12. The reported impression was a grade 1 spondylosis at L4-L5 secondary to facet degenerative change, with mild spinal stenosis.

**Figure 2:**
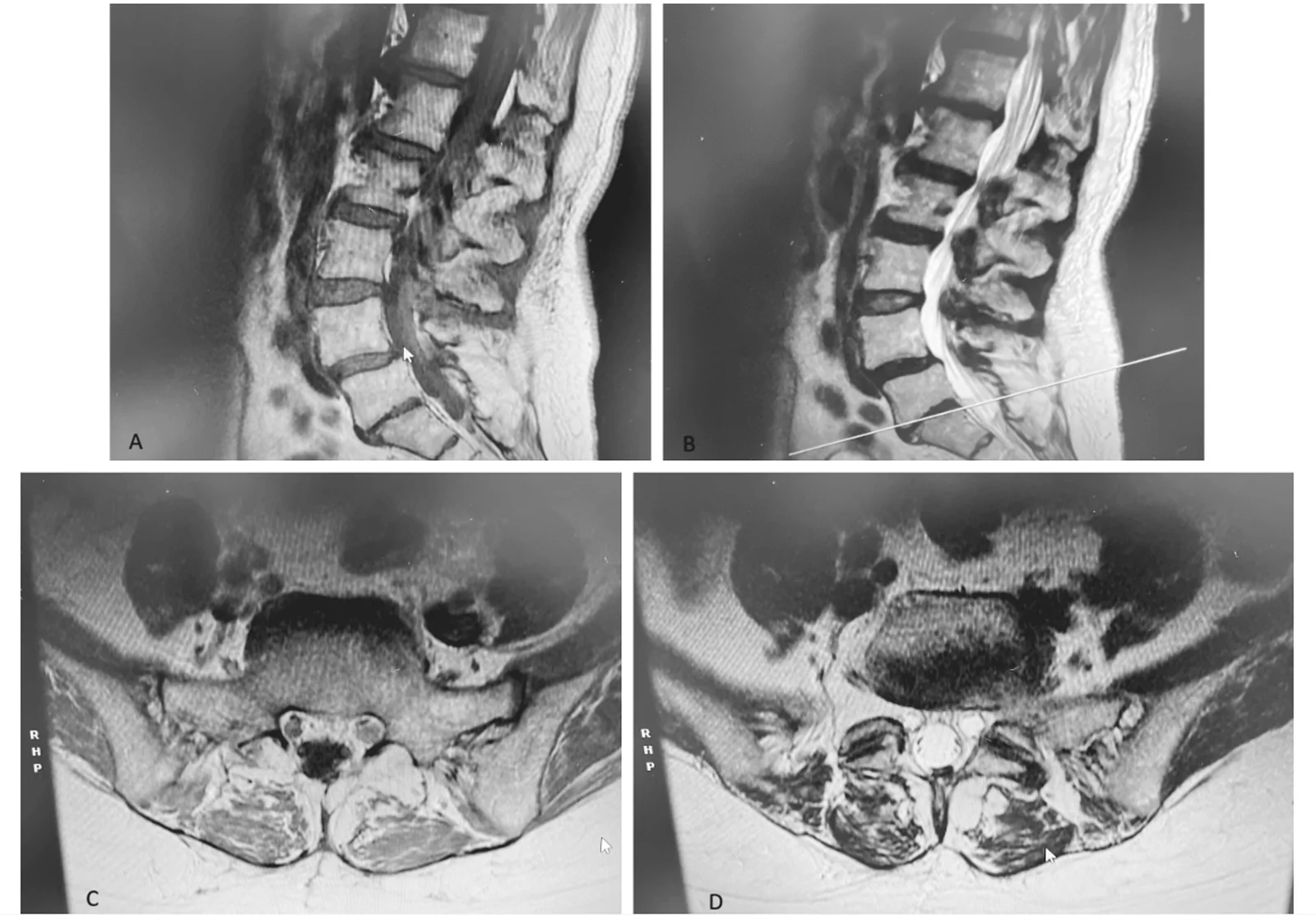
De-identified magnetic resonance images from the lumbar study of September 2014, performed at 1.5 T with and without intravenous gadolinium. Top row, sagittal images: (A) T1-weighted and (B) T2-weighted images demonstrating anterior subluxation of L4 on L5 and multilevel degenerative change. Bottom row, axial images at the L4-L5 level: (C) T1-weighted and (D) T2-weighted images demonstrating severe facet degenerative change, greater on the left, with mild posterolateral encroachment on the dural sac.

Four standing long-cassette or scoliosis-series radiographs were obtained over the next nine years (Table 1 and Figure 3). The 2016 study reported a 35° left-convex lumbar curve measured from the superior endplate of T12 to the inferior endplate of L3, with a 15° right-convex thoracic curve from T7 to T12. The 2018 study reported essentially stable values: a 34° thoracolumbar curve (T12-L3) and a 13° thoracic curve (T7-T11). The 2022 standing long-stitched series reported a 43° lumbar levorotoscoliosis (measured from L1 to L4) and a 25° thoracic dextroscoliosis (T7-L1), together with new mild anterolisthesis of C3-C4 and C4-C5 and persistent mild L4-L5 anterolisthesis. The most recent 2025 study described a 41° lumbar rotoscoliosis with apex at L1, and a 31° thoracic curve with apex at T10, and the reporting radiologist characterized the overall appearance as “severe S-shaped scoliotic curvature... similar to prior study.”

**Table 1:** Standing scoliosis series radiographs (2016-2025). Each study was reported by a different radiologist (Readers 1-4). End vertebrae are reported as they appeared in the original radiology report. Inconsistent end-vertebra selection across studies confounds strict numerical comparison of curve magnitudes (see Discussion). The April 2001 preoperative film (Figure 1) is excluded because its coned lumbar field of view is not comparable with these full-length studies. DDD: degenerative disc disease.

Date	Reader	Thoracic curve (end vertebrae)	Lumbar curve (end vertebrae)	Additional findings
Aug 2016	Reader 1	15° dextro (T7-T12)	35° levo (T12-L3)	
Dec 2018	Reader 2	13° dextro (T7-T11)	34° levo (T12-L3)	
Aug 2022	Reader 3	25° dextro (T7-L1)	43° levoroto (L1-L4)	Mild anterolisthesis C3-C4, C4-5, L4-L5; DDD L1-L2, L4-L5, L5-S1
Jul 2025	Reader 4	31° (apex T10)	41° rotoscoliosis (apex L1)	“Severe S-shaped curvature; similar to prior”

**Figure 3:**
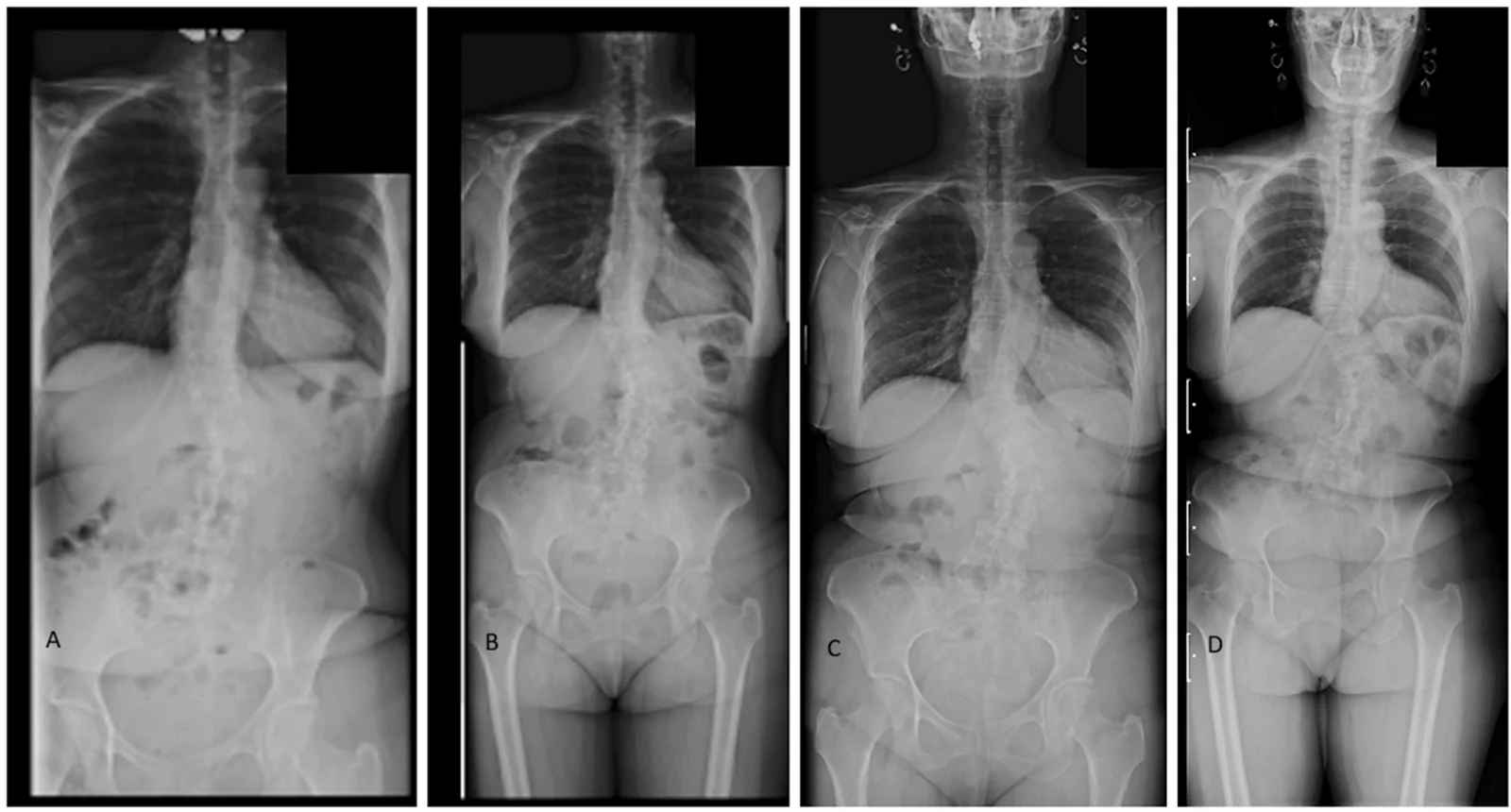
De-identified standing posteroanterior radiographs of the index patient across the nine-year postoperative imaging series, arranged left-to-right by year: panel (A) 2016, panel (B) 2018, panel (C) 2022 (long-stitched long-cassette), panel (D) 2025. All panels normalized to the same height. Progression of the lumbar coronal curve and emergence of a compensatory thoracic curve are visible across the series; see Table 1 for measured Cobb angles and end-vertebra anchors. The preoperative baseline is shown separately in Figure 1. Radiopaque identifying annotation present in the upper outer corner of the original images has been redacted; no anatomy of interest is obscured.

Table 1 summarizes the four standing scoliosis-series studies. Figure 3 shows the corresponding standing AP films, arranged left-to-right by year. The 2001 preoperative film is presented separately as Figure 1 and is deliberately excluded from Table 1, because its limited field of view and non-scoliosis-series technique make its curve magnitude non-comparable with the later studies.

Importantly, the four reports did not use a common pair of end vertebrae for the lumbar curve. The 2016 and 2018 studies anchored the lumbar curve at T12-L3, the 2022 study at L1-L4, and the 2025 study described the curve only by apex (L1). End-vertebra selection is itself a dominant source of inter-rater variability in Cobb measurement [10,11]. Direct numerical comparison of the lumbar angles across the four studies therefore confounds true curve change with measurement noise. With that caveat, the trajectory 145 was progressive: a lumbar curve in the 30s evolved into a curve in the low 40s, with a new compensatory thoracic curve approximately doubling in magnitude.

Management

Based on the clinical examination, the 2014 MRI findings, and standing radiographs, the patient was offered a lumbar facet block in 2016. The procedure was performed after standard informed consent and pre-procedural anticoagulant and nonsteroidal anti-inflammatory drug (NSAID) precautions. Subsequent management consisted of ongoing pharmacological therapy and activity modification. The patient declined further interventional escalation at that time. Over the subsequent years of the treating physician’s care, follow-up was characterized by stable neurological examinations and continued, slowly progressive axial pain without new radicular or myelopathic features.

## Discussion

Mechanisms of post-laminectomy spinal deformity

The patient’s coexisting L4-L5 degenerative spondylolisthesis with severe facet arthropathy, the patient's history of remote lumbar decompressive surgery, and the slow emergence of a progressive coronal deformity together illustrate a pattern that has been described in the post-laminectomy population. Laminectomy without arthrodesis can be followed by radiographic instability at the operated segment: in a cohort of 124 patients followed for a mean of 5.8 years, progressive anterior subluxation occurred in 13% after one-level decompression and in 53% and 59% after two- and three-level decompression, respectively, with incidence rising further with the extent of bone removal [8]. Subsequent systematic review and case-series work have confirmed that new spondylolisthesis can emerge years after isolated decompression, including in patients without overt preoperative instability, with risk modulated by the extent of facet decompression, the number of levels decompressed, and preoperative disc-space height [6,7]. In children and young adults, long-term radiographic follow-up after laminectomy for intraspinal tumors has demonstrated progressive scoliosis and kyphosis at the operated levels [12]; in the adult lumbar spine, an analogous biomechanical logic, i.e., loss of posterior tension-band integrity and accelerated anterior-column degeneration, has been proposed as a substrate for adjacent-segment disease and de novo deformity [13].

The availability of a preoperative radiograph in this case is unusual and bears on the mechanistic question. Because the April 2001 film shows a grossly straight lumbar spine in the coronal plane seven months before surgery, the deformity documented from 2016 onward developed after the decompression rather than preceding it. This ordering is consistent with a post-laminectomy contribution and makes it unlikely that the decompression was performed on an already-deformed spine. It does not, however, establish causation: the patient was approximately 41 years old at the time of surgery and passed through the sixth decade, when de novo degenerative scoliosis characteristically emerges, during the unimaged interval between 2001 and 2016. The two mechanisms remain not mutually exclusive, and the 15-year imaging gap means the tempo and onset of the deformity within that window are unknown. The descriptive radiographic signature of degenerative lumbar deformity, including asymmetric disc degeneration, vertebral body wedging, facet arthropathy, and progressive coronal deformity, is well characterized in the de novo population [3], and we suggest that this phenotype is largely shared by post-laminectomy decompensation, although the two populations have not been formally compared in the literature.

Cobb-angle progression in the context of adult degenerative scoliosis

In a historical cohort of 58 non-surgically managed adult spinal deformity patients followed for a mean of 59.8 months, mean coronal curve progression was 0.83 ± 1.1°/year, with progression observed in 72% of patients and wide individual variation [4]. That same cohort reported a non-significant trend toward faster progression in the de novo degenerative subgroup than in adult idiopathic scoliosis (0.98 ± 1.1 vs. 0.37 ± 1.4°/year; P = 0.07), so the degenerative subgroup rate may be the more apposite comparator for the present patient, albeit on underpowered subgroup data [4]. Community-based work identifies female sex, advancing age, and asymmetric lumbar degeneration as further risk factors [14]. A systematic review identified increasing intervertebral disc degeneration, lateral vertebral translation of ≥6 mm, and an intercrest line passing through L5 as prognostic factors [5]; the same group’s subsequent empirical cohort did not confirm the intercrest line association [4], although it did not retest disc degeneration or lateral translation, so that particular predictor should be regarded as unsettled.

The present patient’s documented lumbar progression, taking the 2016 (35°) and 2022 (43°) measurements at face value, corresponds to an apparent rate of approximately 1.3°/year: above the pooled cohort mean of 0.83°/year and modestly above the de novo degenerative subgroup mean of 0.98 ± 1.1°/year, placing her in that cohort’s highest progression stratum (≥1°/year) [4]. Given the measurement caveats discussed below, this rate should be regarded as an estimate rather than a precise observation. It should also be noted that the comparator cohort excluded patients with any history of spine surgery [4], so it is an imperfect reference population for a post-laminectomy patient.

Measurement variability across serial imaging

A central methodological lesson from this case is the difficulty of comparing Cobb angles obtained across different facilities, radiologists, and protocols over many years. Reported reliability for the Cobb angle is high in formal reproducibility studies, with intra-observer intra-class correlation coefficients above 0.9, inter-observer intra-class correlation coefficients approaching 0.9, and mean inter-observer absolute differences of approximately 3° [15]. A systematic review of Cobb measurement procedures places the limits of agreement at approximately ±2° to ±3°, with mean absolute differences approaching 4° [16]; the 5° threshold conventionally used to define clinically meaningful progression sits just above that envelope. However, those reliability studies typically constrain raters to use the same end vertebrae. When end-vertebra selection is left to the individual reader, as in routine clinical practice, error rates rise substantially, with one recent analysis reporting upper-end vertebra mis-selection in nearly half of curves [10], and end-vertebra ambiguity has long been identified as a dominant source of inter-rater disagreement even when method is otherwise concordant [11].

The four reports in this case used different lumbar end vertebrae (T12-L3 in 2016 and 2018, L1-L4 in 2022, and apex-only description in 2025). Strict comparison of the angles is therefore complicated, and the 2001 preoperative film compounds the problem by using a different field of view and technique altogether. Standardized low-dose biplanar standing radiography and pre-specified Cobb measurement protocols meaningfully reduce this confound. Where such systems are unavailable, an explicit pre-defined measurement protocol, specifying end vertebrae and apex, is a low-cost safeguard for adult deformity surveillance.

Conservative, interventional, and surgical thresholds

Conservative and interventional pain management remained appropriate first-line strategies in this patient given the absence of progressive neurological deficit and her preference to avoid major reconstructive surgery. The lumbar facet block performed in 2016 reflected then-current practice for facet-mediated axial low back pain, supported at the time by narrative reviews and a substantial body of observational evidence, although that review was itself critical of the strength of that evidence base [9]. Subsequent multispecialty consensus guidelines have formalized the role of diagnostic medial branch blocks and radiofrequency neurotomy in selected patients [17]. More recently, a rapid recommendations clinical practice guideline issued strong recommendations against facet joint injections and radiofrequency neurotomy for chronic axial spine pain on the basis of network meta-analyses [18], a recommendation that has generated debate within the interventional pain community and that retrospectively raises the bar for repeating such interventions in patients with limited durable response.

Should conservative care become inadequate, the Scoliosis Research Society (SRS)-Schwab adult spinal deformity classification provides a framework for surgical decision-making that integrates coronal curve type with sagittal-plane modifiers, including pelvic incidence to lumbar lordosis (PI-LL) mismatch, sagittal vertical axis (SVA), and pelvic tilt (PT), each of which correlates with health-related quality of life [19]. Propensity-matched cohort data show meaningfully greater two-year improvement with operative treatment than with continued non-operative care [20], although surgical reconstruction carries substantial risk in older patients. The present patient’s sagittal parameters (pelvic incidence (PI), lumbar lordosis (LL), PI-LL, SVA, PT) were not formally reported on any of the four scoliosis series, and the 2001 film does not permit their retrospective measurement; in our clinical experience, such measurements are an essential prerequisite for a surgical conversation in this population.

Limitations

This is a single-patient case study and inherits the usual limitations of a single observation: the trajectory described here may not generalize to other post-laminectomy patients. The surgical date is confirmed from records, but the operative report could not be retrieved given the interval since surgery and is not expected to become available, so the nature and levels of the 2001 procedure remain permanently inferred and the attribution to laminectomy specifically is provisional; no imaging in this series independently corroborates the procedure type. The preoperative status and date of the April 2001 radiograph rest on the clinical record rather than on the image, which carries no embedded annotation. That radiograph is a coned lumbar view rather than a standing long-cassette series; it indicates the absence of a significant lumbar coronal deformity but cannot exclude a thoracic curve, cannot provide sagittal or pelvic parameters, and has not yet been formally measured. A 15-year imaging gap separates the 2001 film from the 2016 series, so the onset and tempo of deformity within that interval are unknown. The four serial postoperative studies used different end-vertebra anchors, which complicates strict longitudinal numerical comparison as discussed above. None of the four standing series included formal measurement of sagittal pelvic parameters, which limits a complete SRS-Schwab classification and any quantitative surgical-risk assessment in this report. Clinical follow-up data between the imaging studies were not exhaustively reviewed for this report.

## Conclusions

Adult spinal deformity arising in the setting of remote lumbar decompression is a slow-tempo but consequential clinical entity. In this patient, a radiograph obtained seven months before surgery showed a grossly straight lumbar spine, and nine years of subsequent standing radiographs documented progression of a lumbar coronal curve from 35° to the low 40s and the emergence of a compensatory thoracic curve approximately doubling in magnitude, against a background of L4-L5 degenerative spondylolisthesis with severe facet arthropathy. The case highlights three practical points for the clinician managing post-laminectomy patients with axial pain: first, that curve progression can occur in the absence of new neurological symptoms and may not be detected without periodic standing long-cassette imaging; second, that real-world Cobb measurement is confounded by inconsistent end-vertebra selection and benefits from a pre-specified protocol; and third, the threshold for revisiting the conservative-versus-reconstructive decision should be informed by validated deformity classifications, sagittal parameters, and patient-specific factors rather than by curve magnitude alone. Where preoperative imaging can be retrieved, it is worth the effort: it may be the only evidence bearing on whether a deformity followed or preceded decompression, although it establishes sequence rather than cause.
